# Clinical Time Features and Chest Imaging of 85 Patients With COVID-19 in Zhuhai, China

**DOI:** 10.3389/fmed.2020.00209

**Published:** 2020-05-08

**Authors:** Zhuobing Liu, Li Ding, Gongqi Chen, Chaohui Zhao, Xiaoqing Luo, Xinghua Li, Wentao Luo, Jinyu Xia, Xi Liu

**Affiliations:** ^1^Department of Infectious Diseases, The Fifth Affiliated Hospital, Sun Yat-sen University, Zhuhai, China; ^2^Department of Hospital Infection Control, The Fifth Affiliated Hospital, Sun Yat-sen University, Zhuhai, China

**Keywords:** COVID-19, incubation period, clinical characteristics, CT imaging, risk factor

## Abstract

**Background:** An outbreak of SARS-CoV-2 infections began in Wuhan, China, and quickly spread to the entire country. We sought to delineate the time features of clinical symptoms, virological conversion, and chest radiological abnormalities in individuals infected with this virus in Zhuhai, China.

**Methods:** In this retrospective study, we assessed 85 confirmed cases of COVID-19 in the Fifth Hospital of Sun Yat-sen University, Zhuhai, from the 17th of January to the 11th of February 2020. Outcomes were followed up until the 24th of February 2020.

**Results:** The median age of the 85 patients with COVID-19 was 43 years (range, 1–80); 56.5% (48/85) were female. The median time from the last known contact to the first SARS-CoV-2 positive test result was 8 days (0–18). The time to throat swab negativity for SARS-CoV-2 ranged from 5 to 36 days after illness onset. Patients with abnormal chest imaging findings on admission were older than those with normal imaging findings (median age, 50 [3-80] vs. 37 [1-69], *P* = 0.031). Among patients with lung changes on admission, the risk of lesions was 13.8 times greater in the left lower lobe than in the right middle lobe. Most lung lesions appeared within 2 weeks of onset (median 4–5 days). The overall rates of lesions in the right upper/middle/lower lobe and left upper/lower lobe were 47.1, 30.6, 62.4% as well as 49.4 and 63.5%, respectively.

**Conclusions:** The incubation period of SARS-CoV-2 may be longer than 14 days; thus, medical surveillance after contact is required for longer than this. The predominant sites of lung lesions are both lower lungs, whereas the lowest risk region is the right middle lobe.

## Introduction

Beginning in December 2019, a series of patients with acute respiratory disease were presented to health practitioners in Wuhan, Hubei Province, China. The Chinese Centers for Disease Control and Prevention immediately launched the “pneumonia of unknown etiology” mechanism. On the 7th of January 2020, a novel coronavirus named “2019 Novel Coronavirus (2019-NCoV)” was isolated from a patient samples and officially named Severe Acute Respiratory Syndrome Coronavirus 2 (SARS-CoV-2) by the International Committee for Taxonomy of Virus on the 11th of February 2020 (1). SARS-CoV-2 is a β coronavirus. Its genetic signatures differ significantly from those of human Severe Acute Respiratory Syndrome-related coronavirus (SARSr-CoV) and Middle East Respiratory Syndrome-related coronavirus (MERSr-CoV) (2).

On the 22nd of January 2020, the National Health Commission listed Novel Coronavirus Pneumonia as a Class B infectious disease as stipulated in the “Law of the People's Republic of China on the Prevention and Treatment of Infectious Diseases,” and they instituted prevention and control measures for Class A infectious diseases. Wuhan is a transportation hub in central China; thus, infection with SARS-CoV-2 spread quickly to other Chinese cities and many countries worldwide. On the 30th of January 2020, the World Health Organization (WHO) declared this coronavirus outbreak as an “international public health emergency.” As of the 24th of February 2020, there were 77,779 confirmed cases of coronavirus disease (COVID-19) with 2,666 deaths worldwide. The prospects for preventing an epidemic were grim. By this time, the number of infections had far exceeded SARS and MERS, and SARS-CoV-2 appeared to be more contagious, the estimated R0 being 2.2 (3).

The average incubation periods of SARS and MERS are 4.0 and 5.5 days, respectively. In the early stages of the COVID-19 outbreak, experts used these data for SARS and MERS as a reference and estimated the incubation period for COVID-19 to be 2–14 days (4, 5). However, Zhong et al. reported a patient whose reverse-transcriptase polymerase-chain-reaction (RT-PCR) result became positive on the 24th day after known contact (6). Furthermore, 9/80 patients' diagnoses were unconfirmed until their third nucleic acid test (7). Thus, the optimal duration of isolation is still unknown. The typical chest computed tomography (CT) imaging features of COVID-19 pneumonia are multiple patchy ground glass opacities in multiple lobules bilaterally with a peripheral distribution (8). The imaging findings are normal in some patients with early stage infection, pulmonary abnormalities developing as the disease progresses (9). However, the rate of imaging changes in the lungs and the risk of lesions in each lobe are unclear. Compared with the cases in Hubei province, affected patients outside Hubei, China, have exhibited mild or moderate symptoms. There are few published studies on the epidemiological and clinical characteristics and chest imaging findings of patients infected with COVID-19, especially in areas outside Hubei province. We therefore performed this retrospective study to provide more data about COVID-19, including the timeline for clinical symptoms and virological conversion and chest radiological abnormalities.

## Methods

### Ethics Approval

This study was approved by the Institutional Review Board of the Fifth Affiliated Hospital of Sun Yat-sen University (Zhuhai, China) (No. ZDWY [2020] Lunzi No. [K22-1]). The need for consent was waived given the observational and retrospective nature of the study.

### Data Collection

We retrospectively analyzed data of 87 patients with COVID-19 hospitalized in the Fifth Hospital of Sun Yat-sen University, Zhuhai, China, from the 17th of January to the 24th of February 2020. We diagnosed COVID-19 in accordance with the criteria in the WHO interim guidelines and the Diagnosis and Treatment Plan for Novel Coronavirus Pneumonia by the National Health Commission (fifth trial version). A confirmed case was defined as a positive result for throat swab specimens on high-throughput sequencing or real-time RT-PCR assay. These findings were rechecked by the Zhuhai Center for Disease Control in Guangdong Province. By detailed review of the electronic medical records, we collected information, such as the patient's epidemiological history, time of last contact (time of last contact with a known infected individual or of leaving Hubei Province), date of onset of disease (day when symptoms first noticed), time of attending a clinic, admission time, laboratory test positive and negative conversion times, chest radiological abnormalities, and time to improvement. Follow-up lasted until the 24th of February 2020. Two experienced radiologists independently evaluated all CT data.

### Statistical Analysis

Quantitative data were analyzed by using by Pearson's χ^2^ or Fisher's exact test for discrete variables where appropriate. *P*-values < 0.05 were considered to denote significant differences. All tests were two-tailed, and associations were assessed with odds ratios (OR) and 95% confidence intervals (CIs). All analyzes were performed using SPSS software (release 25.0), GraphPad Prism 8, and Microsoft EXCEL.

## Results

### Study Patients

Two of the 87 hospitalized patients with COVID-19 were excluded because they had not undergone CT imaging. One, a 77-year-old man, was in a critical condition and died 32 days after admission. The other was too young to undergo a CT scan. The median age of the remaining 85 enrolled patients was 43 years (range, 1–80), 56.5% (48/85) were female, and 78.8% (67/85) of patients or their family members had visited Hubei Province within 14 days of onset of disease. One or more comorbidities were present in 43.5% (37/85) patients, the commonest being cardiovascular disease (45.9%, 17/37), endocrine disease (24.3%, 9/37), respiratory disease (10.8%, 4/37), and malignant tumors (10.8%, 4/37). On admission, 22 patients had mild infections, 56 moderate, and seven severe (Table 1).

**Table 1:** Baseline characteristics of SARS-CoV-2-infected patients on admission.

		**CT imaging**		
	**Total (*N* = 85)**	**Abnormal (*N* = 61)**	**Normal (*N* = 24)**	***P*-value**
**Age, years**	43	50	37	0.031
Sex, Female	48 (56.5%)	35 (57.4%)	13 (54.2%)	0.788
**Comorbidities**				
Cardiovascular diseases	17 (45.9%)	16 (26.2%)	1 (4.2%)	0.058
Endocrine disease	9 (24.3%)	8 (13.1%)	1 (4.2%)	0.458
Respiratory diseases	4 (10.8%)	3 (4.9%)	1 (4.2%)	1.000
Malignant tumor	4 (10.8%)	3 (4.9%)	1 (4.2%)	1.000
Digestive tract disease	3 (8.1%)	3 (4.9%)	0 (0%)	0.560
Smoking history	3 (8.1%)	2(3.3%)	1(4.2%)	1.000
**Case classification**				
Mild	22 (25.9%)	0 (0%)	22 (91.7%)	Reference
Moderate	56 (65.9%)	54 (88.5%)	2 (8.3%)	0.000
Severe	7 (8.2%)	7 (11.5%)	0 (0%)	0.000
**Number of lobes involved**				
1	13 (15.3%)	13 (21.3%)	0	–
2	13 (15.3%)	13 (21.3%)	0	–
3	14 (16.5%)	14 (23.0%)	0	–
4	8 (9.4%)	8 (13.1%)	0	–
5	13 (15.3%)	13 (21.3%)	0	–
**Cure out of hospital**	54 (63.5%)	41 (67.2%)	13 (54.2%)	0.855

**Figure 1:**
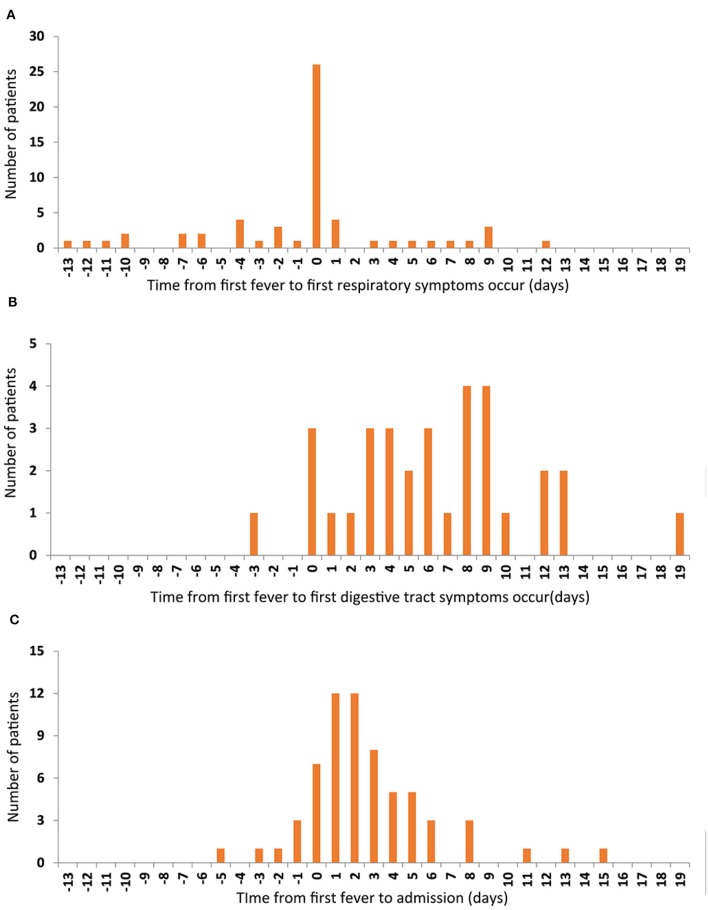
The relationship among the first onset of the three symptoms. The number of patients are represented on the y-axis. The time from first fever to first respiratory symptoms occur **(A)**, to first digestive tract symptoms occur **(B)**, and to admission time **(C)** are represented on the x-axis.

The commonest symptoms throughout the disease were fever (75.3%, 64/85), cough (55.3%, 47/85), and sore throat (23.5%, 20/85), and the less frequently occurring symptoms were fatigue (12.9%, 11/85), muscular soreness (12.9%, 11/85), and headache (10.6%, 9/85). The least common symptoms were diarrhea, chest distress, nausea, or/and vomiting. Seven individuals were completely asymptomatic (Table 2). The times of onset of fever, respiratory symptoms, and digestive tract symptoms were collected, and it was found that fever and respiratory symptoms often occurred simultaneously (Figure 1A), whereas digestive tract symptoms appeared later than fever (Figure 1B). Most patients were admitted to hospital within 1 week of onset of fever (Figure 1C).

**Table 2:** Symptoms of SARS-CoV-2-infected patients throughout the disease.

**Symptoms**	**Total (*N* = 85)**	**CT imaging on admission**		***P* value**	**CT imaging throughout the disease**		***P* value**
		**Abnormal (*N* = 61)**	**Normal (*N* = 24)**		**Abnormal (*N* = 71)**	**Normal (*N* = 14)**	
		***n* (%)**	***n* (%)**		***n* (%)**	***n* (%)**	
Fever	64 (75.3%)	49 (80.3%)	15 (62.5%)	0.147	57 (80.3%)	7 (50.0%)	0.016
Cough	47 (55.3%)	33 (54.1%)	14 (58.3%)	0.724	39 (54.9%)	8 (57.1%)	0.879
Pharyngalgia	20 (23.5%)	15 (24.6%)	5 (20.8%)	0.713	17 (23.9%)	3 (21.4%)	1.000
Muscular soreness	11 (12.9%)	9 (14.8%)	2 (8.3%)	0.664	9 (12.7%)	2 (14.3%)	0.664
Fatigue	11 (12.9%)	8 (13.1%)	3 (12.5%)	1.000	9 (12.7%)	2 (14.3%)	0.664
Headache	9 (10.6%)	5 (8.2%)	4 (16.7%)	0.453	7 (9.9%)	2 (14.3%)	0.638
Anhelation	7 (8.2%)	6 (9.8%)	1 (4.2%)	0.676	6 (8.5%)	1 (7.1%)	0.676
Diarrhea	5 (5.9%)	2 (3.3%)	3 (12.5%)	0.288	3 (4.2%)	2 (14.3%)	0.188
Chest distress	4 (4.7%)	3 (4.9%)	1 (4.2%)	1.000	4 (5.6%)	0	–
Nausea or/and Vomiting	3 (3.5%)	2 (3.3%)	1 (4.2%)	1.000	2 (2.8%)	1 (7.1%)	1.000
Asymptomatic	7 (8.2%)	5 (8.2%)	2 (8.3%)	1.000	5 (7.0%)	2 (14.3%)	0.324

### Times to Diagnosis and Viral Clearance

**Figure 2:**
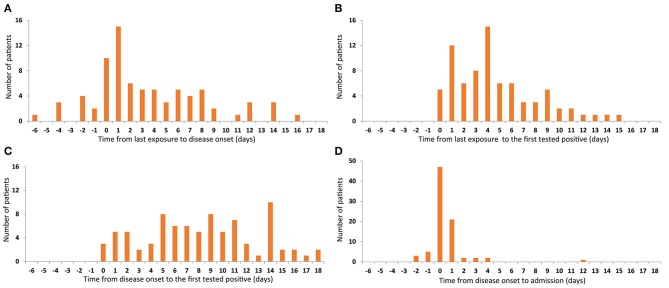
Time features related to the first tested positive. Number of patients are represented on the y-axis. Time from last exposure to disease onset are represented on the x-axis **(A)**. Time from last exposure **(B)**, disease onset **(C)**, and admission **(D)** to the first positive RT-PCR throat swab are represented on the x-axis.

The median time from the last exposure to the onset of disease was 2 days (−6 to 16). Symptoms developed within 1 week after the last contact in 74.1% (63/85) of patients and 11.8% (10/85) left the epidemic area/confirmed patient after onset of symptoms (Figure 2A). The median time between onset of symptoms and the first RT-PCR-positive throat swab was 4 days (0–15), RT-PCR-positivity occurring within 1 week of the onset of symptoms in most cases (Figure 2B). The median time from the last contact to the first RT-PCR positive throat swab was 8 days (0–18), 90.6% (77/85) of patients having positive RT-PCR tests within 14 days of leaving the epidemic area or confirmed patient(s), and 21.2% (18/85) of patients were completely asymptomatic or had very mild symptoms. Most of these 18 patients were diagnosed during observation as inpatients because they were close contacts. The longest interval from contact to RT-PCR-positivity was 18 days, which may mean that the incubation period of SARS-CoV-2 is longer than is currently believed. A longer isolation time is therefore required (Figure 2C). The median time from admission to the first positive RT-PCR throat swab was 0 days (−2 to 12). Although one patient was first found to be RT-PCR-positive on the 12th day of admission, 89.4% (76/85) patients tested positive for RT-PCR within 1 day of admission (Figure 2D), indicating that the scope of screening needs to be extended to maximize identification of asymptomatic carriers.

**Figure 3:**
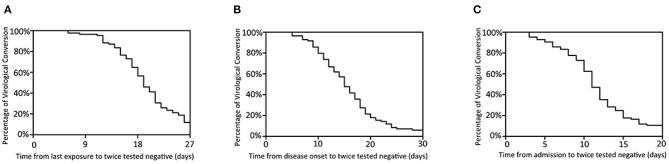
Time features of viral clearance. Percentage of virological conversion are represented on the y-axis. The time from last exposure **(A)**, disease onset **(B)**, admission **(C)** to twice negative RT-PCR throat swab are represented on the x-axis.

Twice negative RT-PCR tests is one of the criteria for lifting of quarantine. At the time of final follow-up, 80 patients had met this criterion. The median times from last contact, onset, and admission to twice negative RT-PCR tests were 19 days (6–38), 15 days (5–36), and 11 days (3–26), respectively. Most patients had recovered and were discharged in the second week after admission (Figure 3). Of the remaining five, the longest hospitalization was 35 days, the other four all being hospitalized for more than 2 weeks.

### Characteristic Chest CT Imaging Findings

In addition to RT-PCR, CT is an essential component of evaluations. Chest CTs generally showed small patchy shadows and interstitial lung disease, which further developed into ground glass attenuation and infiltration. Pulmonary consolidation occurred in severely affected patients; however, pleural effusion was relatively rare.

**Figure 4:**
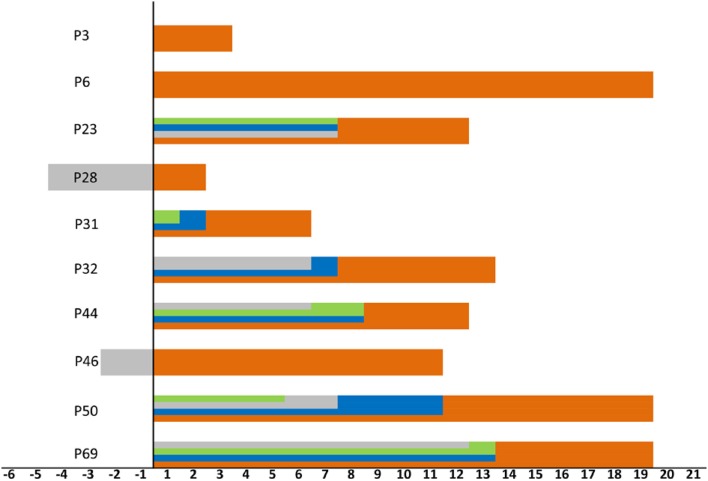
Timeline for the 10 patients encountered lung lesions after admission. Zero means last contact, distinct periods are shown as onset (gray), isolation (green), admission (blue), and first abnormal CT findings (orange). The time of onset, isolation, admission, and last contact of the patient 3 and 6 were on the same day. The time of isolation, admission and last contact of the patient 28 and 46 were on the same day.

Abnormal chest imaging findings were present at the time of admission in 71.8% (61/85) patients, those with abnormalities being significantly older than those with normal CT scans on admission (median age, 50 [3-80] vs. 37 [1-69], *P* = 0.031). Patients encountered fever could be a predictor of abnormal chest imaging findings (*P* = 0.016, Table 2). Comorbidities tended to be present more frequently in those with chest CT abnormalities than in those without them (cardiovascular disease 16 [26.2%] vs. one [4.2%], endocrine disease eight [13.1%] vs. one [4.2%], respiratory disease three [4.9%] vs. one [4.2%], digestive tract disease three [4.9%] vs. none [0%], and malignant tumor three [4.9%] vs. one [4.2%]; however, these differences were not statistically significant (Table 1). In addition, 10 of the 24 patients with normal imaging findings on admission developed pulmonary abnormalities on CT as the disease progressed, such patients being older than those with persistently normal chest CT scans (median age, 38 [33–69] vs. 29 [1-65] years, *P* = 0.089). When we examined the time intervals between the last contact of these 10 patients and onset (gray), isolation (green), admission (blue), and first abnormal CT findings (orange), we found that the last contact, isolation, and admission of four of them were on the same day (Figure 4).

**Figure 5:**
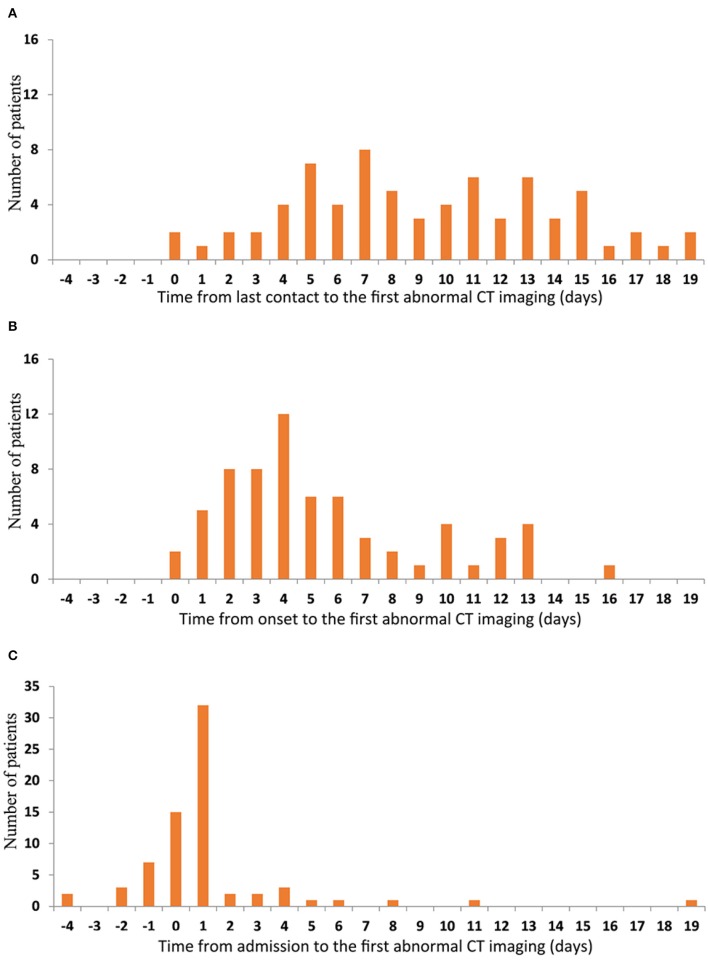
Time features of the first abnormal CT imaging. The number of patients are represented on the y-axis. The time from last contact **(A)** onset **(B)** and admission **(C)** to the first abnormal CT imaging are represented on the x-axis.

The median times from the last contact, onset, and admission to the first abnormal CT scan in the 71 patients who had abnormal CT scans at some stage were 8 days (0–19), 4 days (0–16), and 1 day (−4–19), respectively. Imaging abnormalities developed at varying intervals after the last contact with no clear peak in timing (Figure 5A). Most patients had imaging abnormalities within 1 week of onset of the disease (Figure 5B). A few had imaging abnormalities before admission and the vast majority were found to have lung lesions within 2 days of admission; however, one patient did not show imaging changes until 19 days after admission (Figure 5C).

There were no significant differences in the number of infected lobes in patients with imaging abnormalities on admission (Table 1). Analysis of patterns of distribution in the affected lungs of 61 patients on admission revealed that the probability of lesions was lowest in the right middle lobe. The risks of infection in the right upper, right lower, left upper, and left lower lobes were 2.5, 4.9, 3.4, and 13.8 times higher, respectively, than the risk of infection in the right middle lobe. The left lower lobe was the most frequently involved, its rate of infection being 4.0, 5.6, and 2.8 times higher than that of the left upper, right upper, and right lower lobes, respectively (Table 3).

**Table 3:** Comparison of the risk of lesion in different lobes of the lung of 61 patients with abnormal CT imaging on admission.

**Diseased region**	**Abnormal**	**Normal**	***P*-value**	**OR (95% CI)**
Right middle lobe	18 (29.5%)	43 (70.5%)	Reference	
Right upper lobe	31 (50.8%)	30 (49.2%)	0.016	2.469 (1.172~5.199)
Right lower lobe	41 (67.2%)	20 (32.8%)	0.000	4.897 (2.274~10.547)
Left upper lobe	36 (59.0%)	25 (41.0%)	0.001	3.440 (1.624~7.285)
Left lower lobe	52 (85.2%)	9 (14.8%)	0.000	13.802 (5.632~33.825)
Right upper lobe	31 (50.8%)	30 (49.2%)	Reference	
Right lower lobe	41 (67.2%)	20 (32.8%)	0.066	1.984 (0.953~4.130)
Left upper lobe	36 (59.0%)	25 (41.0%)	0.363	1.394 (0.681~2.851)
Left lower lobe	52 (85.2%)	9 (14.8%)	0.000	5.591 (2.348~13.314)
Left upper lob	36 (59.0%)	25 (41.0%)	Reference	
Right lower lobe	41 (67.2%)	20 (32.8%)	0.348	1.424 (0.680~2.981)
Left lower lobe	52 (85.2%)	9 (14.8%)	0.001	4.012 (1.677~9.600)
Right lower lobe	41 (67.2%)	20 (32.8%)	Reference	
Left lower lobe	52 (85.2%)	9 (14.8%)	0.019	2.818 (1.161~6.842)

The risk of lesions in the right and left lower lobes did not differ significantly in the 85 patients on admission, the rates of lesions in the right upper/middle/lower lobe and left upper/lower lobe being 36.5, 21.2, and 48.2% as well as 42.4 and 61.2%, respectively. Up to 24 February, the endpoint of our study, the predominant site of lung lesions was the left lower lobe (63.5%). However, this rate did not differ significantly from that of the right lower (62.4%) or left upper lobe (49.4%). The risk of lesions in the right middle lobe was still the lowest (Table 4).

**Table 4:** Comparison of the Risk of lesion in different lobes of the lung of 85 COVID-2019 patients.

**Diseased region**	**On admission**				**Time of data collection (Feb 24)**			
	**Abnormal**	**Normal**	***P*-value**	**OR (95% CI)**	**Abnormal**	**Normal**	***P*-value**	**OR (95% CI)**
Right middle lobe	18 (21.2%)	67 (78.8%)	Reference		26 (30.6%)	59 (49.4%)	Reference	
Right upper lobe	31 (36.5%)	54 (63.5%)	0.028	2.137 (1.080~4.228)	40 (47.1%)	45 (52.94%)	0.028	2.017 (1.077~3.779)
Right lower lobe	41 (48.2%)	44 (81.8%)	0.000	3.468 (1.771~6.793)	53 (62.4%)	32 (37.6%)	0.000	3.758 (1.988~7.104)
Left upper lobe	36 (42.4%)	49 (57.6%)	0.003	2.735 (1.392~5.372)	42 (49.4%)	43 (50.6%)	0.012	2.216 (1.184~4.151)
Left lower lobe	52 (61.2%)	33 (38.8%)	0.000	5.865 (2.974~11.566)	54 (63.5%)	31 (36.5%)	0.000	3.953 (2.087~7.487)
Right upper lobe	31 (36.5%)	54 (63.5%)	Reference		40 (47.1%)	45 (52.94%)	Reference	
Right lower lobe	41 (48.2%)	44 (81.8%)	0.121	1.623 (0.879~2.997)	53 (62.4%)	32 (37.6%)	0.045	1.863 (1.011~3.434)
Left upper lobe	36 (42.4%)	49 (57.6%)	0.433	1.280 (0.691~2.371)	42 (49.4%)	43 (50.6%)	0.759	1.099 (0.602~2.006)
Left lower lobe	52 (61.2%)	33 (38.8%)	0.001	2.745 (1.475~5.106)	54 (63.5%)	31 (36.5%)	0.031	1.960 (1.061~3.620)
Left upper lob	36 (42.4%)	49 (57.6%)	Reference		42 (49.4%)	43 (50.6%)	Reference	
Right lower lobe	41 (48.2%)	44 (81.8%)	0.441	1.268 (0.693~2.323)	53 (62.4%)	32 (37.6%)	0.436	1.262 (0.703~2.266)
Left lower lobe	52 (61.2%)	33 (38.8%)	0.014	2.145 (1.162~3.958)	54 (63.5%)	31 (36.5%)	0.063	1.783 (0.966~3.292)
Right lower lobe	41 (48.2%)	44 (81.8%)	Reference		53 (62.4%)	32 (37.6%)	Reference	
Left lower lobe	52 (61.2%)	33 (38.8%)	0.090	1.691 (0.919~3.110)	54 (63.5%)	31 (36.5%)	0.874	1.052 (0.564~1.960)

**Figure 6:**
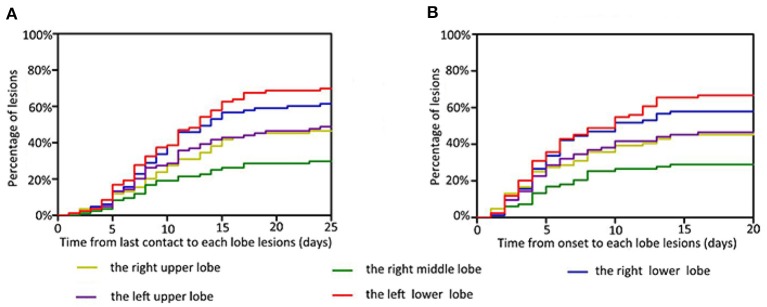
Time features related to the infection of each lobe. Infection risk in the right middle lobe is the lowest, in the left lower lobe is the highest, and in both upper lungs is similar. The percentage of lesions are represented on the y-axis. The time from last contact **(A)** and onset **(B)** to each lobe lesions are represented on the x-axis.

When we examined the time from last exposure to detection of lesions in each lobe, we found that the median interval was 9 days for the right upper lobe (0–23), 8 days for the right middle lobe (0–23), 8 days for the right lower lobe (0–24), 8 days for the left upper lobe (0–24), and 8 days for the left lower lobe (0–24). Most patients showed imaging changes in the second week after the last contact (Figure 6A). The median times from onset to detection of lesions in each lung lobe were 4 days for the right upper lobe (0–20), 4 days for the right middle lobe (0–14), 4 days for the right lower lobe (0–14), 4 days for the left upper lobe (0–16), and 5 days for the left lower lobe (0–16); thus, most imaging abnormalities appeared within 1 week of the onset of symptoms (Figure 6B). One patient showed imaging changes 24 days after the last contact.

## Discussion

The incubation period is important in diagnosis and control of infectious diseases. The most recent study reported a mean incubation period of 5.2 days (95% confidence interval [CI], 4.1–7.0), the 95th percentile of the distribution being 12.5 days. We recommend that the duration of quarantine should be at least 14 days on the basis of the 95th percentile estimate of the incubation period (10). In our study, one asymptomatic patient tested positive for viral nucleic acid as long as 18 days after leaving Hubei, indicating that patients with SARS-CoV-2 may need to be isolated for longer.

In our study, achievement of twice negative RT-PCR throat swabs ranged from 5 to 36 days after onset. The possibility of reverting to positive after viral conversion has not been ruled out. Thus far, we do not have a clear understanding of this aspect of the etiology of SARS-CoV-2. Given that the time to MERS-CoV negativity among survivors ranges from 1 to 44 days from illness onset (11), it may be necessary to set a longer period of follow-up.

In our study, the proportion of patients with fever at any stage of the disease was 75.3% (64/85), which is similar to that reported from Jiangsu (22 January to 24 February 2020; 78.8%, 63/80) (7) and Guangzhou (from 26 January to 4 February 2020; 78%, 70/90) (8). All of these rates are lower than that reported from Wuhan, Hubei province, which was 83–98.6% (12, 13). One possible explanation for this discrepancy may be that the outbreak in Wuhan was the earliest and most serious, the research period being during January. The disease spread to other areas from around the end of January to February. Because medical departments were by then alert to the importance of extensive screening and early detection and isolation of infected individuals, these patients' symptoms were mild at the time of admission to hospital. Furthermore, 3.5% (3/85) of patients in our study only had digestive tract symptoms, such as nausea, vomiting, and diarrhea, without any respiratory symptoms or fever on admission. Of individuals who were screened because they were close contacts, 8.2% (7/85) had a concealed onset without no obvious symptoms.

Our previous study showed that viral loads can be detected in asymptomatic patients, indicating that asymptomatic patients may also be infectious and are likely to be highly contagious in the early stages of infection (14). Thus, in contrast to SARS, these characteristics indicate the need to expand the scope of screening and screen earlier, thereby ensuring accurate identification and management of patients in the early stages of disease progression and preventing a potential pandemic in the absence of a vaccine or treatment.

Chest CT imaging is also very useful in early detection of suspected cases. The typical imaging manifestations of COVID-19 are similar to those of SARS, namely, ground glass opacities in the lung parenchyma in the early stages and areas of consolidation in the later stages, some of which are round, the lesions are mostly distributed on the periphery of the lung (15). On admission, 71.8% (61/85) patients had abnormal imaging findings. These patients were older than the other 28.2% (24/85) patients who had normal CT scans (*P* = 0.031). However, we detected no predilection for male vs. female patients, and found no differences in other clinical features between the two groups. Pathological changes can occur in both lungs, mainly in both lower lobes, and the risk of infection in the left lower lobe is 13.8 times higher than that in the right middle lobe. This conclusion is consistent with Salehi's research (16), and we speculate that it may be due to the physiological structure of the right middle lobe. In addition, whether there is a difference in gene expression in the right middle lobe leads to different susceptibility, which requires further study of pathological data and in-depth study of the mechanism.

As the course of the disease progressed, 10/24 (41.7%) patients developed new lung lesions, and the risk of infection is highest in the left lower lobe and lowest in the right middle lobe. By dynamically observing changes in CT findings, we found that lesions characteristically appeared first in the lower lung and developed upward, which may explain the late appearance of upper respiratory symptoms after exposure to the source of infection. Possibly because our study cohort is too small, our findings do not reflect the time differences in imaging lesions. It is also possible that some patients were in clusters, as having experienced close contact between an initially diagnosed patient being found to have pulmonary lesions on CT scans. The fact that pulmonary lesions do not appear soon after exposure does not mean indicate that SARS-COV-2 has a short incubation period.

Within a week of onset, 50 of our patients had abnormal chest CT scans, whereas 61 had positive RT-PCT tests, suggesting that RT-PCR detects SARS-CoV-2 earlier than chest CT imaging.

This study has several limitations. First, no CT scans were performed before admission; thus, lung lesions may appear earlier than we detected. Second, as we had a small cohort of patients and short follow-up, our conclusions need to be further verified by large samples and multi-center data.

In conclusion, in this observational study, the incubation period of SARS-CoV-2 was found to sometimes exceed 14 days, indicating the need for more prolonged surveillance. Most lung lesions appear within 2 weeks of onset, the median interval being 4–5 days. The lesions were predominantly in both lower lungs, the risk of lesions being lowest for the right middle lobe. Further in-depth study of patients with COVID-19 is still needed.

## Data Availability Statement

The datasets for this manuscript are not publicly available because the datasets include the patients' information, which could not be made publicly available online. Requests to access the datasets should be directed to Xi Liu, liuxi26@mail.sysu.edu.cn.

## Ethics Statement

This study was approved by the institutional review board of the Fifth Affiliated Hospital of Sun Yat-sen University (Zhuhai, P.R. China) (No. ZDWY[2020] Lunzi No. [K22-1]). Waiver of consent was obtained given the observational nature of the project.

## Author Contributions

JX and XLiu designed the study. ZL and LD wrote the manuscript. GC, CZ, and XLu attended patients and provided clinical data. XLi and WL performed data analysis.

## Conflict of Interest

The authors declare that the research was conducted in the absence of any commercial or financial relationships that could be construed as a potential conflict of interest.
